# History of Preeclampsia Adds to the Deleterious Effect of Chronic Stress on the Cardiac Ability to Flexibly Adapt to Challenge

**DOI:** 10.3389/fphys.2018.01237

**Published:** 2018-09-03

**Authors:** Helmut K. Lackner, Manfred G. Moertl, Karin Schmid-Zalaudek, Miha Lucovnik, Elisabeth M. Weiss, Vassiliki Kolovetsiou-Kreiner, Ilona Papousek

**Affiliations:** ^1^Section of Physiology, Otto Loewi Research Center, Medical University of Graz, Graz, Austria; ^2^Department of Obstetrics and Gynecology, Clinical Center, Klagenfurt, Austria; ^3^Department of Perinatology, Division of Obstetrics and Gynecology, University Medical Centre Ljubljana, Ljubljana, Slovenia; ^4^Institute of Psychology, Biological Psychology Unit, University of Graz, Graz, Austria; ^5^Department of Obstetrics and Gynecology, Medical University of Graz, Graz, Austria

**Keywords:** blunted cardiac reactivity, pregnancy complications, cardiovascular adaptations, cardiovascular complications, perceived stress, acute challenges

## Abstract

Preeclampsia, a pregnancy-specific disorder, presents a major health problem during gestation, but is also associated with increased risk for cardiovascular complications in later life. We aimed to investigate whether chronic stress experience and preeclampsia may have additive adverse effects on the cardiac ability to flexibly adapt to challenge, that is, to mount an appropriately vigorous heart rate response to an acute psychological challenge, or whether they may perhaps have synergistic effects (e.g., mutual augmentation of effects). Blunted cardiac responding to challenge has been linked to poor health outcomes in the longer term. Women previously affected by preeclampsia and women after uncomplicated pregnancies were tested 15–17 weeks post-partum in a standardized stress-reactivity protocol, while cardiovascular variables were simultaneously recorded. Changes in heart rate and blood pressure in response to the stressor were analyzed with regard to the effects of history of preeclampsia and chronic stress experience. Findings indicated blunted cardiac responses in women with higher chronic stress experience (*p* = 0.020) and, independently from that, in women with a history of preeclampsia (*p* = 0.018), pointing to an additive nature of the effects of preeclampsia and chronic stress on impaired cardiovascular functioning. Consequently, if both are present, a history of preeclampsia may add to the already deleterious effects of the experience of chronic stress. The additive nature of the effects suggests that stress-reducing interventions, albeit they will not eliminate the heightened cardiovascular risk in patients with a history of preeclampsia, may improve their overall prognosis by avoiding further accumulation of risk.

## Introduction

Preeclampsia is a pregnancy-specific disorder characterized by sudden onset of hypertension and proteinuria after the 20th week of gestation, occurring in 2–8% of pregnancies (Duley, 2009). It is associated with conditions that are potentially life threatening for the mother and fetus. There is substantial evidence that preeclampsia does not only present major health problems during the pregnancy period, but is also associated with increased risk for cardiovascular complications in later life (Brown et al., 2013; Wu et al., 2017).

It has been debated whether preeclampsia may be an independent risk factor for later cardiovascular disease and add to an already elevated risk (Craici et al., 2008; Brown et al., 2013; Wu et al., 2017) or an early marker of an *a priori* high-risk profile (Sattar and Greer, 2002; Craici et al., 2008). Consequently, several authors argued for continuing monitoring of cardiovascular risk factors in women with a history of preeclampsia (Ray et al., 2005; Craici et al., 2008; Wu et al., 2017). Perhaps even more important, preeclampsia may offer the chance to become aware of increased risk at an early enough stage for women to benefit from intervention such as modification of their lifestyle (Sattar and Greer, 2002; Williams, 2003).

Identifying factors that are compromised in women with preeclampsia and at the same time play some role in the multifactorial process promoting cardiovascular disease is essential for the development of efficient intervention programs. Pregnancy can be considered as a “stress test” of the somatic and cardiovascular system. On the one hand, pregnancy is associated with huge cardiovascular and metabolic changes resembling a metabolic syndrome including relative insulin resistance, hyperlipidaemia and increase in coagulation (Sattar and Greer, 2002; Williams, 2003; Craici et al., 2008; Bilhartz et al., 2011). Preeclampsia, in which typical autonomically regulated cardiovascular adaptations to pregnancy are absent (Lugue et al., 2016) may indicate “failing the stress-test,” and may, therefore, be predictive of future cardiovascular disorders when similar adverse factors will be present. While in later life these may be less dramatic than during pregnancy, they may on the other hand more persisting and, thus, negatively affect health in the longer term.

On the other hand, due to challenges to adapt to various psychosocial and physiological changes, the time of pregnancy is also a huge stressor in the psychological sense (Woods et al., 2010; Guardino and Schetter, 2014). Symptoms of depression and anxiety, prevalent in prenatal phases and post-natally (O’Hara et al., 1991; Bennett et al., 2004; Parfitt and Ayers, 2014; Schofield et al., 2014) indicate that some women “fail the stress-test” (Guardino and Schetter, 2014), especially when symptoms are persistent (Fredriksen et al., 2017) or manifested as post-traumatic stress symptoms. Threatening conditions such as preeclampsia potentiate the distress and entail post-traumatic stress symptoms in a substantial number of women (Engelhard et al., 2002; Hoedjes et al., 2011; Stramrood et al., 2011).

Inability to effectively cope with the challenges of life is linked to chronic stress experience, which refers to the appraisal of demands as taxing or exceeding the resources of the individual and is relatively independent from the objective characteristics of the situation itself (Lazarus and Folkman, 1984; Lazarus, 1993; Ellsworth and Scherer, 2003). It is well established that chronic stress experience is associated with heightened risk for cardiovascular complications (Rosengren et al., 2004; Richardson et al., 2012; Steptoe and Kivimäki, 2013). Importantly, there is emerging evidence that women are even more vulnerable to the adverse effects of stress perceptions on the risk of cardiovascular complications than men (Vaccarino and Bremner, 2017).

While it may take decades for systemic changes to erupt in a cardiovascular incident, more immediate effects of chronic stress experience on the cardiovascular system manifest in blunted cardiac responses to acute challenges (Allen et al., 2011; Ginty and Conklin, 2011; Hughes et al., 2011; Brindle et al., 2013). Blunted cardiac reactivity to challenges refers to a lower than typically observed cardiac response to standard stressful laboratory tasks that require active coping such as socially evaluative cognitive performance tasks (e.g., mental arithmetic, public speaking). It reflects an inability to mobilize the functional, beta-adrenergically mediated cardiac response to effectively cope with challenges (McEwen, 1998; Richter et al., 2008; Carroll et al., 2017), and occurs despite rating the challenge as equally or even more stressful (Salomon et al., 2009; Bibbey et al., 2013; Brindle et al., 2017). The concept is similar to the concept of “chronotropic incompetence,” which has emerged in the context of physical exercise testing (Ellestad and Wan, 1975).

The association between chronic stress experience and impaired cardiac ability to flexibly adapt to challenge tends to be stronger in women than in men (Allen et al., 2011). Attenuated heart rate responses to standard cognitive tasks were also reported in patients with post-traumatic stress disorder (Peckerman et al., 2003). One study focusing on a different research question more incidentally reported attenuated heart rate responses to an acute cognitive challenge in post-menopausal women with a history of (mixed) hypertensive pregnancies compared to women with normotensive pregnancies 40 years after childbirth. Chronic stress experience did not differ between groups at the time of testing.

While in earlier research mainly excessive reactivity was discussed as a factor in the development of cardiovascular complications (Gerin et al., 2000; Schwartz et al., 2003), more recent accounts increasingly focus on the importance of attenuated cardiac reactivity to acute challenges. Previous studies indicate that blunted cardiac reactivity to acute mental challenge is associated with a variety of poor health outcomes (for review see Phillips et al., 2013). More specifically, attenuated heart rate reactivity to mental stress tasks was associated with a higher risk of clinical cardiovascular events among patients who had undergone recent bypass surgery (Herd et al., 2003). Several large prospective studies demonstrated that individuals without cardiovascular disease at the time of testing who showed attenuated heart rate increases during exercise had higher incidences of developing cardiovascular disease and mortality during the following years (Sandvik et al., 1995; Lauer et al., 1996, 1999; Gulati et al., 2010). Additionally, evidence suggests that attenuated heart rate reactivity during exercise is more prognostically important in women than in men (Daugherty et al., 2011).

The aim of the present study was to investigate whether chronic stress experience and preeclampsia may have additive adverse effects on the cardiac ability to flexibly adapt to challenge, that is, to mount an appropriately vigorous heart rate response to an acute psychological challenge, or whether they may perhaps even have synergistic effects (e.g., such that effects of chronic stress experience are larger in preeclamptic patients than in controls or vice versa). Women with a history of preeclampsia and mothers with uncomplicated pregnancies were tested in a controlled experiment 15 to 17 weeks post-partum. They were exposed to a standardized memory task for testing their heart rate reactivity to acute challenge, and their chronic stress experience was assessed using a standard psychometric instrument.

## Materials and Methods

### Participants

In two large hospitals, eligible women with a history of preeclampsia and women with uncomplicated pregnancies, preselected on the basis of their medical records were invited to participate in the study. Preeclampsia was confirmed using the recommendations of the American College of Obstetricians and Gynecologists (2013). Task Force on Hypertension in Pregnancy. Inclusion criteria were: Systolic blood pressure ≥ 140 mmHg and/or diastolic blood pressure ≥ 90 mmHg, present ≥ 20 weeks’ gestation and return to normotensive values within 12 weeks post-partum, blood pressure measured twice, at least 4 h apart. Proteinuria: either Protein ≥ 300 mg per 24-h urine collection, or Protein/creatinine ratio ≥ 0.3, or Protein ≥ 30 mg/dl or 1+ on urine dipstick. Participants in the control group had uncomplicated singleton pregnancies with term delivery. Exclusion criteria in both groups were: diabetes mellitus, renal disease, chronic hypertension, antiphospholipid antibody syndrome, kidney transplant, hypothyroidism, thyroid antibodies, pre-existing cardiovascular problems, seizures. Women with multiple gestation, or substance abuse (alcohol, tobacco, illegal drugs) were also excluded. In addition, in both groups appropriate education (minimum school-leaving qualification) and language competence (native German or German B2) were required for inclusion. A total of 30 women with a history of preeclampsia and 40 women with uncomplicated pregnancies were finally included in the study. Nine out of the 30 women with a history of preeclampsia had early-onset preeclampsia (< week 34 of gestation; Tranquilli, 2013), 19 (including all patients with early-onset preeclampsia) were diagnosed with severe preeclampsia according to recommended definitions (Tranquilli, 2013). Five fetuses were diagnosed with suspected intrauterine growth restriction (IUGR), but none reached the WHO (1995) criterion of birth weight below the 10^th^ percentile for the gestational age. All participants provided informed written consent under ethical approval granted by the local Ethics committee, Medical University Graz (No. 27–515 ex 14/15) and the Ethics committee Carinthia (No. A16/15). **Table 1** presents an overview of demographic, basic cardiovascular, and clinical characteristics of the study sample.

**Table 1:** Demographic, basic cardiovascular and clinical characteristics of the study sample.

	History of preeclampsia *n* = 30	Uncomplicated pregnancy *n* = 40	*p*-value
Age [years]	33.9 ± 5.0, 25–42	31.7 ± 4.5, 21–44	*p* = 0.064
**Level of education**			*p* = 0.981
Less than high school (*n*)	5	6	
High school graduate (*n*)	9	12	
Some college (*n*)	16	22	
BMI [kg/m^2^]	26.8 ± 5.4, 20–41	24.8 ± 4.5, 17–36	*p* = 0.092
Depression CES-D	8.8 ± 6.8, 1–26	8.5 ± 5.8, 0–24	*p* = 0.847
Social support ESSID	23.7 ± 1.6, 20–25	23.9 ± 1.5, 19–25	*p* = 0.701
**Cardiovascular values at rest**			
Heart rate [bpm]	71.4 ± 8.5, 51–86	72.1 ± 7.7, 59-89	*p* = 0.686
Systolic BP [mmHg]	111.7 ± 11.1, 88–130	107.1 ± 9.3, 92-130	*p* = 0.068
Diastolic BP [mmHg]	73.9 ± 9.2, 58–93	68.9 ± 8.1, 50-87	*p* < 0.05
**Medication^+^**			
Antihypertensive medication	12	0	
Acetylsalicylic acid	6	1	
Delivery (day of gestation)	253 ± 19, 211–287	278 ± 9, 254–293	*p* < 0.001
Baby weight [g]	2553 ± 806, 1245–3940	3415 ± 334, 2780–4250	*p* < 0.001
Baby height [cm]	47 ± 4, 41–57	51 ± 2, 47–60	*p* < 0.001
**Birth procedure**			*p* < 0.001
Spontaneous delivery	6	30	
Cesarean section	20	5	
Vacuum extraction	4	5	

*Note: Metric values are represented as mean ±*SD*, min – max (range); + the medication was taken during gestation only*.

### Procedure

Participants were asked to participate in the study 13–15 weeks post-partum and were tested 15–17 weeks after delivery. The experiment started with an approximately 30-min period in which the participating women could adapt and settle down. In this period general questions were asked, electrodes were attached and the electrophysiological signals were checked. Furthermore, the participants filled in the Perceived Stress Questionnaire to measure chronic stress, that is, subjectively experienced stress independent of a specific and objective occasion (Fliege et al., 2005) and the depression scale (Hautzinger and Bailer, 1993) as well as some other questionnaires that are not relevant to this paper. After a 5-min resting period in which the participants were asked to remain seated, not to speak, and to relax, the memory task was explained using a prerecorded auditory instruction backed by corresponding information on a computer screen. To increase the self-relevance of the task and, hence, its stressful character, participants were told that physical changes that occur during pregnancy may also affect functioning of the brain. As mnemonic abilities are a particular sensitive indicator for such decrements, their test performance will be evaluated by colleags from the psychiatry department who will determine whether their mnemonic abilities are corresponding to their age or indicate premature aging of the brain. The task was to recall as many words as they could from a list of words that will be read out to them (cf. Gendolla, 1999). Items of the memory task were 16 words from a standardized memory test (California Verbal Learning Test, German adaptation; Niemann et al., 2008). After they had confirmed that they had understood the instruction, the prerecorded word list was delivered via headphones (20 s). Then, a 30 s preparation period followed in which a numerical counter on the computer screen counted down from 30 to 1, and participants were finally prompted to start reproducing the words. Participants were given a minimum of 30 s to recall and recite as many words as they could. After 30 s had elapsed, they were asked if they had recited all words they could recall and were given some more time if they had not. Following completion of the memory task, there was another 5-min relaxation period, and participants rated how difficult and how stressful they had perceived the task on two 17-point rating scales (ranging from “not difficult at all” to “extremely difficult” and from “not stressful at all” to “extremely stressful,” cf. Papousek et al., 2011, 2017; Lackner et al., 2015). Participants remained seated during the entire study protocol.

### Recording and Quantification of Cardiovascular Variables

Continuous monitoring of heart rate and blood pressure was carried out with the Task Force Monitor^®^ (CNSystems, Graz, STY, Austria) throughout the whole procedure. Heart rate was recorded by 3-lead electrocardiography (ECG; sampling rate = 1 kHz) using CNSystems ECG-electrodes placed at the thoracic region. The continuous blood pressure (sampling rate = 100 Hz) was derived from the finger using a refined version of the vascular unloading technique and corrected to absolute values with oscillometric blood pressure measurement on the contralateral upper arm (Fortin et al., 2006).

The study protocol was controlled in a fully automated way using proven software-tools developed in MATLAB^®^ (MathWorks Natick, Massachusetts, MA, United States) (Lackner et al., 2013, 2014). For the present study the cardiovascular data of the four periods (initial resting period, task preparation, word list recall and final resting period) and the change scores (word list recall – initial resting period), (task preparation – initial resting period) were analyzed.

Heart rate changes during the preparation period preceding the actual task were analyzed in addition to those during word list recall, because anticipation of an upcoming cognitive challenge elicits cardiac responses comparable to those during execution of the task, with the advantage that they are not contaminated with the effects of behavioral components (such as speaking) and additional uncontrolled psychological components (such as the perceived quality of task performance). This anticipatory activation is considered to reflect preparatory mobilization of metabolic resources for appropriate responding to the upcoming demands (Obrist, 1981; Brehm and Self, 1989).

### Statistical Analysis

The main research question was evaluated with two multiple regression analyses, one with the heart rate change from rest to word list recall, and one with the heart rate change from rest to task preparation as the dependent variable. History of preeclampsia (preeclampsia vs. uncomplicated pregnancy), the level of chronic stress experience, and the rating how stressful the task was perceived were entered as the predictors. The latter variable was included in order to remove any inter-individual differences in the perception of the task, which allows a more unequivocal interpretation of the two main variables of interest (history of preeclampsia, chronic stress experience). In step two of the regression analyses, the interaction term of chronic stress experience by history of preeclampsia was added to the regression equation to test for a potential synergistic effect of the two variables.

The overall effect of the challenge was tested with a oneway repeated measures analysis of variance with heart rate during the initial resting period, task preparation, word list recall, and the final resting period as the dependent variable. A supplementary analysis tested for differences in task performance between the two groups (number of correctly recalled words) as an objective indicator of task effort (independent *t*-test). Further supplementary analyses were done to explore potential differences in the perceived difficulty of the task, age, BMI, depression, day of gestation, basic blood pressure and heart rate, baby weight and height (independent *t*-tests), and level of education and birth procedure (Chi-square test). Finally, supplementary regression analyses analogous to those described above were conducted with changes in systolic and diastolic blood pressure as the dependent variables. Descriptive data are reported as mean ± standard deviation. A significance level of alpha = 0.05 (two-tailed) was used for all analyses. Effect sizes of statistically significant results are reported as η^2^ (analysis of variance, *t*-tests) and *sr*^2^ (regression analyses). Both scores indicate how much variance of the dependent variable is explained by a specific independent variable, independently of the other independent variables in the analysis (proportion of uniquely explained variance).

## Results

**FIGURE 1:**
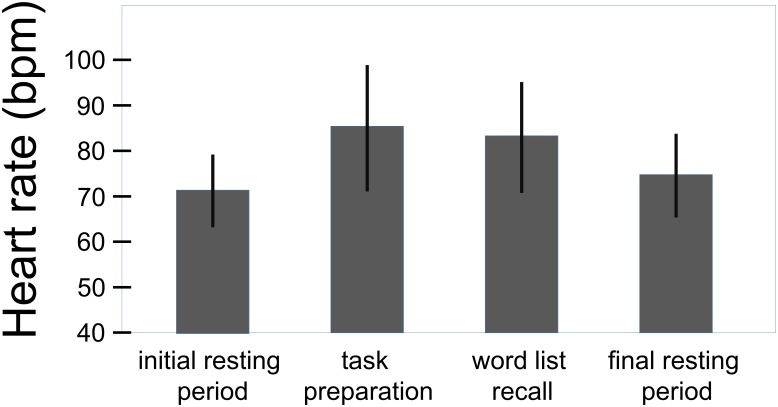
Overall effect of the challenge on the heart rate (total sample, *n* = 70). Whiskers represent standard deviations. (*F*(3,207) = 76.8, *p* < 0.001, η^2^ = 0.527).

**Figure 1** shows the large overall effect of the challenge in terms of heart rate changes. Heart rate increased from 71.8 ± 8.0 bpm to 85.4 ± 13.9 bpm from rest to task preparation and to 83.3 ± 12.7 bpm during word list recall; after completion of the memory test it returned to 74.8 ± 9.2 bpm (*F*(3,207) = 76.8, *p* < .001, η^2^ = 0.527). Basic heart rate (initial resting condition) of women with a history of preeclampsia did not differ from that of mothers with uncomplicated pregnancies (*t*(68) = 0.4, *p* = 0.686).

**FIGURE 2:**
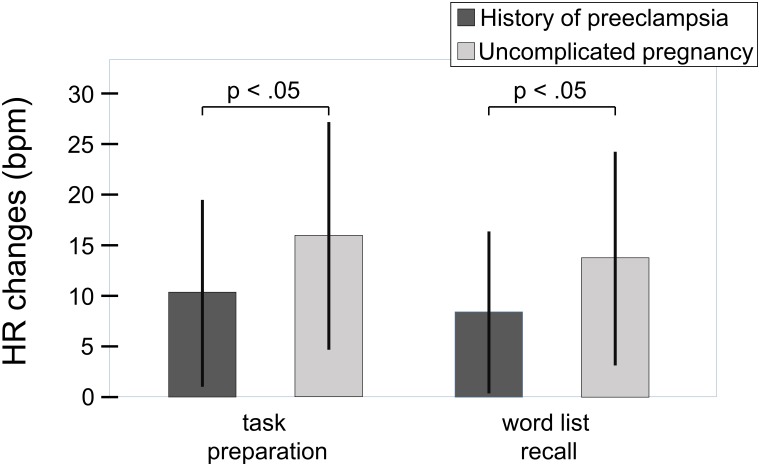
Heart rate responses to the challenge relative to initial resting period in women with history of preeclampsia (*n* = 30) and women with uncomplicated pregnancies (*n* = 40). Whiskers represent standard deviations. Independently from chronic stress experience, women with a history of preeclampsia showed smaller heart rate responses than women with uncomplicated pregnancies (word list recall: *sr* = –0.29, *p* = 0.018; *sr*^2^ = 0.084; *F*(3,66) = 3.9, *p* = 0.013; task preparation: *sr* = –0.28, *p* = 0.020, *sr*^2^ = 0.078; *F*(3,66) = 3.8, *p* = 0.015).

The regression analysis with changes of heart rate from rest to word list recall as the dependent variable (*F*(3,66) = 3.9, *p* = 0.013) revealed independent effects of chronic stress experience (*r* = -0.27, β = -0.27, *sr* = -0.28, *p* = 0.020, *sr*^2^ = 0.078) and history of preeclampsia (*r* = -0.27, β = -0.28, *sr* = -0.29, *p* = 0.018, *sr*^2^ = 0.084). Higher levels of chronic stress experience were linked to attenuated heart rate responses to the challenge. Independently from that, women with a history of preeclampsia showed smaller heart rate responses than women with uncomplicated pregnancies (**Figure 2**). The interaction between the two variables did not explain additional variance of heart rate reactivity to the challenge (*F*_change_(1,67) = 0.62, *p* = 0.435), substantiating the additive nature of the effects of chronic stress experience and history of preeclampsia. The regression analysis with heart rate changes from rest to task preparation as the dependent variable yielded essentially the same results (*F*(3,66) = 3.8, *p* = 0.015; chronic stress experience: *r* = -0.26, β = -0.26, *sr* = -0.27, *p* = 0.028, *sr*^2^ = 0.073; history of preeclampsia: *r* = -0.26, β = -0.27, *sr* = -0.28, *p* = 0.020, *sr*^2^ = 0.078; *F*_change_(1,65) = 1.32, *p* = 0.255). The rating of how stressful the task was perceived did not have a significant effect in either analysis. Heart rate responses to the challenge and the preparatory signal were highly correlated (*r* = 0.89, *p* < 0.001). The statistically significant correlations are considered to be of medium size according to the common conventions of Cohen (1988). Patients with early- vs. late-onset preeclampsia (*t*(28) = 0.44, *p* = 0.662; *t*(28) = 0.87, *p* = 0.392) and with mild vs. severe preeclampsia (*t*(28) = 1.34, *p* = 191; *t*(28) = 1.53, *p* = 0.137) did not differ in their heart rate responses to the performance stress.

**FIGURE 3:**
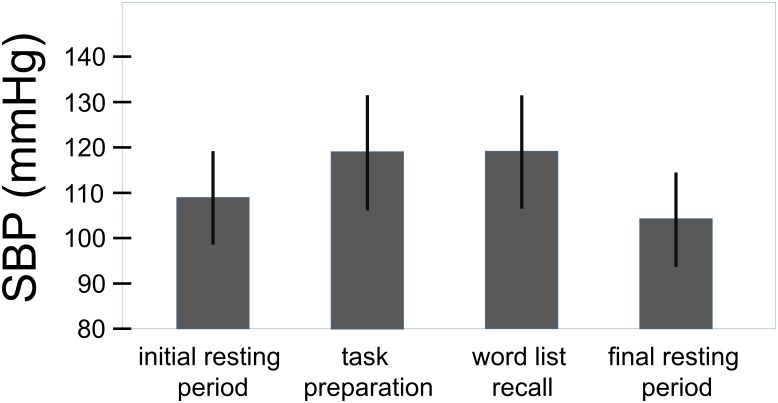
Overall effect of the challenge on the systolic blood pressure (total sample, *n* = 70). Whiskers represent standard deviations. (*F*(3,207) = 59.7, *p* < 0.001, η^2^ = 0.464).

**FIGURE 4:**
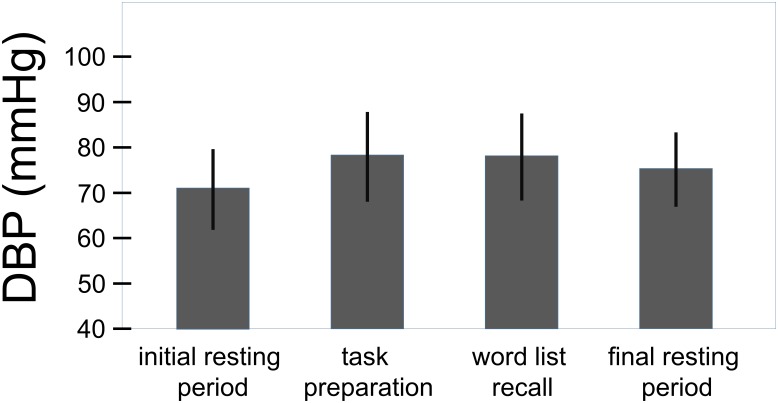
Overall effect of the challenge on the diastolic blood pressure (total sample, *n* = 70). Whiskers represent standard deviations. (*F*(3,207) = 53.4, *p* < 0.001, η^2^ = 0.439).

The overall effect of the challenge on the systolic and the diastolic blood pressure can be seen in **Figures 3**, **4**. For the systolic blood pressure an increase from 109.1 ± 10.3 mmHg to 119.5 ± 12.7 mmHg from rest to task preparation and to 119.9 ± 12.5 mmHg during word list recall; after completion of the memory test it returned to 114.3 ± 10.4 mmHg (*F*(3,207) = 59.7, *p* < 0.001, η^2^ = 0.464). The diastolic blood pressure mirrored this results with 71.1 ± 8.9 mmHg at rest, 78.3 ± 9.9 mmHg during task preparation and 78.1 ± 9.6 mmHg during word list recall; after completion of the memory test the diastolic blood pressure returned to 75.3 ± 8.2 mmHg (*F*(3,207) = 53.4, *p* < 0.001, η^2^ = 0.439). The diastolic blood pressure was higher in women with a history of preeclampsia than in women with uncomplicated pregnancies (diastolic: *t*(68) = -2.41, *p* = 0.019, η^2^ = 0.09, systolic: *t*(68) = -1.85, *p* = 0.068) but systolic and diastolic were well below critical values (see **Table 1**).

Analyses testing for potential effects on blood pressure responses yielded no statistically significant results (word list recall, systolic: *F*(3,66) = 0.22, *p* = 0.885; word list recall, diastolic: *F*(3,66) = 0.24, *p* = 0.871; task preparation, systolic: *F*(3,66) = 0.04, *p* = 0.991; task preparation, diastolic: *F*(3,66) = 0.04, *p* = 0.991). No differences between patients with early- vs. late-onset preeclampsia and with mild vs. severe preeclampsia were observed (all *p* > 0.380).

Task performance did not differ between women with a history of preeclampsia and women with uncomplicated pregnancies (*t*(68) = -0.736, *p* = 0.464). On average, participants correctly recited 7.3 ± 1.6 words (min = 4, max = 11 words). Women with and without a history of preeclampsia rated the task equally difficult (*t*(68) = 0.198, *p* = 0.843) and equally stressful (*t*(68) = -1.089, *p* = 0.280). On the scales ranging from 1 to 17, difficulty ratings were 9.3 ± 3.6, ratings of how stressful the task was were 12.0 ± 3.6.

Participants of the two study groups did not significantly differ in age (*t*(68) = -1.89, *p* = 0.064), body mass index (*t*(68) = -1.71, *p* = 0.092), level of education (χ^2^ = 0.039, *p* = 0.981), depressive symptoms (*t*(68) = 0.19, *p* = 0.847) or social support (*t*(68) = 0.14, *p* = 0.701) As was to be expected, women with a history of preeclampsia gave birth earlier (*t*(68) = 6.78, *p* < 0.001, η^2^ = 0.45), were more likely to have a cesarean section, and their babies were smaller at birth (weight: *t*(68) = 6.19, *p* < 0.001, η^2^ = 0.36; height: *t*(68) = 5.39, *p* < 0.001, η^2^ = 0.30) compared to women with uncomplicated pregnancies (see **Table 1**).

## Discussion

The results of the present study indicate that a history of preeclampsia is associated with difficulties in mounting appropriately sized cardiac responses to acute challenge, thereby impairing the ability to dynamically adapt to environmental challenges. Adaptive biological responses to acute challenges involve brisk increases in activation as the individual mobilizes for active coping (McEwen, 1998). Blunted cardiac reactivity was also observed as a function of chronic stress perception. The assumedly deleterious effects of the experience of chronic stress and of a history of preeclampsia on the cardiac ability to flexibly adapt to challenge are clearly additive. That is, if the system is already affected by chronic stress, a history of preeclampsia adds to the adverse development.

The similarity to the effect of perceived chronic stress suggests that the attenuated heart rate reactivity to acute challenge of women with a history of preeclampsia is a consequence of preeclampsia rather than a pre-existing factor in those at risk for developing preeclampsia. Preeclampsia is an extremely stressful condition, which may elicit serious post-traumatic symptoms in a considerable proportion of affected women (Engelhard et al., 2002; Hoedjes et al., 2011; Stramrood et al., 2011). Blunted heart rate responses to standard laboratory challenges have been observed in individuals who had experienced major life events such as having been the target of violent or sexual offense (Lovallo et al., 2012; Voellmin et al., 2015) and in individuals suffering from post-traumatic stress disorder (Peckerman et al., 2003).

Flexible responding to active performance challenges (i.e., vigorous heart rate increase during the challenge and rapid return to baseline values afterward) depends on the integrity of the direct autonomic innervation of the heart (Shapiro et al., 1993). One possible explanation for the effect of chronic stress or extreme stress such as in preeclampsia on heart rate reactivity to acute challenges is that frequent or sustained activation of sympathetic nerves provoked by the stress may cause down-regulation of beta-adrenergic receptors (reduced receptor density and/or sensitivity) and, consequently, post-synaptic desensitization of the beta-adrenergic receptor pathway in the sino-atrial node. This has been suggested in the context of research focusing on attenuated heart rate reactivity to exercise (Kawasaki et al., 2010; and see also McEwen, 1998). In this case it is also possible that heart rate responses to acute challenges are attenuated despite increased sympathetic activation, because sympathetic activation may not be well translated into the functional response, that is, heart rate increase (Kawasaki et al., 2010). The observation of blunted heart rate responses in users of stimulant medications (Westover et al., 2015) corroborates this idea.

Others suggested that blunted heart rate responses in challenging situations demanding active coping may reflect mainly central nervous system mediated suboptimal functioning (Carroll et al., 2017; see also Lugue et al., 2016). There also seem to exist at least some genetic influences on the cardiac reactivity to active performance tasks. Heritability estimates range from 0.26 to 0.43 (Wu et al., 2010). Without doubt, more research is required to understand the underlying mechanisms for blunted heart rate reactivity in stressed individuals.

The blunted heart rate reactivity in women with a history of preeclampsia was not mirrored in their blood pressure responses to the challenge. Interestingly, a meta-analysis indicated that older individuals show attenuated heart rate reactivity despite higher systolic blood pressure responses during emotionally evocative tasks in laboratory challenges (Uchino et al., 2010). Basically, blood pressure increases in the face of acute psychological stressors are adaptive reactions supporting stress-related behavioral responding (McEwen, 1998; Gianaros and Sheu, 2009; Carroll et al., 2017). However, in addition the interaction between age-related changes in cardiovascular reactivity to stress and social networks were shown (Uchino et al., 2016). In our study, no difference in the blood pressure reactivity during the stressful performance situation between women with a history of preeclampsia and women with uncomplicated pregnancies were seen but no difference in the task performance, the reported social support and depression symptoms were seen as well.

One implication of the present findings may be that women with a history of preeclampsia should be made aware of their potentially increased risk of cardiovascular disease and interventions for improving their stress management should be recommended, in order to avoid further accumulation of risk. While evidence of direct links between blunted heart rate reactivity to laboratory challenges and risk of later cardiovascular complications is relatively sparse (Herd et al., 2003), there is extensive evidence of links to various other adverse health outcomes (Phillips et al., 2013), including stress-related psychiatric disorders such as depression (York et al., 2007; Salomon et al., 2009; Ehrenthal et al., 2010; Phillips et al., 2013), post-traumatic stress disorder (Peckerman et al., 2003), and anxiety (Souza et al., 2015). These, in turn, are predictors of cardiovascular risk on their part, particularly depression (Bunker et al., 2003; Cohen et al., 2015), but also anxiety disorders and post-traumatic stress disorder (Edmondson and Cohen, 2013; Brudey et al., 2015). Women with a history of preeclampsia also tend to have additional risk factors such as a greater body mass index or a more unfavorable lipid profile (Bokslag et al., 2017), which may further contribute to their risk.

In line with the understanding that the appraisal of situations or events as stressful is more important than the objective characteristics of one’s situation (Lazarus and Folkman, 1984; Lazarus, 1993; Ellsworth and Scherer, 2003), blunted cardiac reactivity was particularly observed in individuals who perceived their lives as more stressful than their reported stress exposure would suggest (Ginty and Conklin, 2011). In studies relating objective life events to cardiovascular reactivity, findings have been more mixed (Carroll et al., 2005; Ginty and Conklin, 2011). Thus, interventions for developing strategies for improved stress management seem promising, also if major life events may be present that are not changeable as such. The use of adaptive coping strategies is critical in the susceptibility to stress-induced cardiovascular disease (Wood and Valentino, 2017).

Since hypertensive disorders of pregnancy have become a well-recognized risk factor for future cardiovascular health, several attempts have already been made to include obstetric history in routine screening of cardiovascular risk factors (Spaan et al., 2012). However up to now, very little guidance exists regarding the actual prevention of cardiovascular disease among women with a history of preeclampsia. Strategies to reduce body mass index, to increase physical activity, adherence to the Dietary Approaches to Stop Hypertension (DASH) diet, and low dietary sodium/potassium intake have recently been showed to reduce risk of chronic hypertension in women with history of hypertensive disorders of pregnancy (Moore et al., 2001; Sacks et al., 2001; Adrogué and Madias, 2007; Carnethon et al., 2010; Cornelissen et al., 2011; Landsberg et al., 2013; Timpka et al., 2017). Our study suggests that stress reduction programs should also be considered among such interventions.

A limitation of the present research is that it cannot be generalized to all kinds of demands. While highly reliable within tasks, cardiovascular reactivity may considerably vary across tasks (e.g., active performance tasks vs. emotional stimulation or cold pressure task; Kelsey et al., 2007). Furthermore, it is also important to note that we do not necessarily consider blunted cardiac responses as a direct causal factor in the development of future cardiovascular complications but rather as an indicator that something in the central-autonomic regulation processes is not right. Nevertheless, evidence is emerging that links blunted cardiovascular reactivity to psychological stressors to a variety of poor health outcomes (Phillips et al., 2013). The present study focused on transient changes of heart rate, which index the net effects of fast sympathetic and parasympathetic inputs to the sinus node and represent a sensitive, flexible, and rapidly responding system in the context of psychophysiological adaptations (Goldberger, 1999). Future research may go deeper into the matter and address changes in regulation mechanisms by taking other processes such as the baroreflex or the synchronization among physiological systems into account (e.g., Moertl et al., 2013). Future research in larger samples may also give more attention to the impact of type and severity of the preeclampsia on autonomic nervous system adaptations (e.g., Weber et al., 2017) as well as to potentially important context variables such as intrauterine growth and birth procedure, which may further add to the stress of pregnancy. A further crucial variable to be considered is the time lag with which the measures are taken. Walther et al. (2014), analyzing heart rate variability four days after delivery, concluded that the maternal cardiovascular system was still strongly affected by pregnancy at that time, whereas findings of Kolovetsiou-Kreiner et al. (2018) suggested that 6 weeks post-partum, the autonomic nervous system, biochemical endothelial reactions and pulse transit time showed opposing trends compared to pregnancy findings. In the present study, mothers were tested 15 to 17 weeks post-partum when cardiovascular regulation had largely stabilized and huge fluctuations in basic cardiovascular regulation were no longer to be expected.

To conclude, the present study once more supported the notion that some implications of the pregnancy specific disorder preeclampsia extend to at least post-partum and perhaps even beyond that. However, the relationship of pregnancy complicated by preeclampsia and future cardiovascular risk might also provide a chance for the implementation of primary prevention strategies, including interventions targeting at regular preventive medical check-ups, changes of life-style as well as the improvement of adaptive coping with stress.

## Author Contributions

HL and IP: conception and design of the study, data analysis, interpretation of the data, drafting and revising the manuscript. MM participated in the conception of the study and revised the manuscript critically for important intellectual content. KS-Z and VK-K: acquisition and interpretation of the data, revising the manuscript. ML and EW: interpretation of the data and revising the manuscript critically for important intellectual content. All authors have approved this version submitted for publication and agree to be accountable for all aspects of the work in ensuring that questions related to the accuracy or integrity of any part of the work are appropriately investigated and resolved.

## Conflict of Interest Statement

The authors declare that the research was conducted in the absence of any commercial or financial relationships that could be construed as a potential conflict of interest.
